# Neurophysiological differences between patients clinically at high risk for schizophrenia and neurotypical controls – first steps in development of a biomarker

**DOI:** 10.1186/s12916-015-0516-z

**Published:** 2015-11-02

**Authors:** Frank H. Duffy, Eugene D’Angelo, Alexander Rotenberg, Joseph Gonzalez-Heydrich

**Affiliations:** Department of Neurology, Boston Children’s Hospital and Harvard Medical School, 300 Longwood Ave, Boston, Massachusetts 02115 USA; Department of Psychiatry, Boston Children’s Hospital and Harvard Medical School, 300 Longwood Ave, Boston, Massachusetts 02115 USA

**Keywords:** Biomarker, Clinical high risk, Discriminant function analysis, Electroencephalogram spectral coherence, Frequency modulated auditory evoked response, Principal component analysis, Prodrome, Schizophrenia

## Abstract

**Background:**

Schizophrenia is a severe, disabling and prevalent mental disorder without cure and with a variable, incomplete pharmacotherapeutic response. Prior to onset in adolescence or young adulthood a prodromal period of abnormal symptoms lasting weeks to years has been identified and operationalized as clinically high risk (CHR) for schizophrenia. However, only a minority of subjects prospectively identified with CHR convert to schizophrenia, thereby limiting enthusiasm for early intervention(s). This study utilized objective resting electroencephalogram (EEG) quantification to determine whether CHR constitutes a cohesive entity and an evoked potential to assess CHR cortical auditory processing.

**Methods:**

This study constitutes an EEG-based quantitative neurophysiological comparison between two unmedicated subject groups: 35 neurotypical controls (CON) and 22 CHR patients. After artifact management, principal component analysis (PCA) identified EEG spectral and spectral coherence factors described by associated loading patterns. Discriminant function analysis (DFA) determined factors’ discrimination success between subjects in the CON and CHR groups. Loading patterns on DFA-selected factors described CHR-specific spectral and coherence differences when compared to controls. The frequency modulated auditory evoked response (FMAER) explored functional CON–CHR differences within the superior temporal gyri.

**Results:**

Variable reduction by PCA identified 40 coherence-based factors explaining 77.8 % of the total variance and 40 spectral factors explaining 95.9 % of the variance. DFA demonstrated significant CON–CHR group difference (*P* <0.00001) and successful jackknifed subject classification (CON, 85.7 %; CHR, 86.4 % correct). The population distribution plotted along the canonical discriminant variable was clearly bimodal. Coherence factors delineated loading patterns of altered connectivity primarily involving the bilateral posterior temporal electrodes. However, FMAER analysis showed no CON–CHR group differences.

**Conclusions:**

CHR subjects form a cohesive group, significantly separable from CON subjects by EEG-derived indices. Symptoms of CHR may relate to altered connectivity with the posterior temporal regions but not to primary auditory processing abnormalities within these regions.

## Background

Schizophrenia is a chronic, severe, and disabling mental disorder characterized by deficits in thought processes, perceptions, and emotional responsiveness. Furthermore, it is associated with symptoms including cognitive disorganization, hallucinations, and paranoia. The prevalence per year in the USA is 1.1 % of the adult population (www.nimh.nih.gov/health/topics/schizophrenia).

The yearly incidence for psychotic disorders has been reported recently in Australia as 28 per 100,000 population, with rates peaking in adolescence and young adulthood [1]. However, a longitudinal register-based case finding approach, as opposed to a first-contact sampling approach, clarified that the incidence of schizophrenia is likely many times higher than commonly reported, reaching as high as 69 per 100,000 patients per year in the USA [2].

Medications have failed to ‘cure’ schizophrenia and response to pharmacologic intervention is reported to be quite variable [3], which may relate in part to poor medication compliance [4] and may also have biological underpinnings. For example, 30 % of patients with inadequate medication response had a group-specific magnetic resonance imaging (MRI) pattern of marked frontal atrophy [5]. Many publications suggest that, although schizophrenia cannot be medically ‘cured’, various support strategies including social, psychological, and environmental – as well as pharmacologic – may prove substantially ameliorative and suggest that the course of schizophrenia be considered ‘uneven’ rather than ‘chronic’ [6–8].

Schizophrenia has been increasingly viewed from a developmental perspective. Full psychosis appears to represent a later aspect of the disorder. This raises the possibility that medical and/or pharmacologic interventions provided early might be of greater help to patients in the long term. As reviewed by Larson et al. [3], prior to onset of diagnosed schizophrenia in adolescence or young adulthood, and prior to persistent psychosis, there may be a ‘prodromal’ or clinical high risk (CHR) period, lasting from several weeks to several years, during which aberrant behaviors are evident.

Although the notion of neurodevelopmental influences upon schizophrenia originally arose from postulated changes in fetal brain development [9], more recent thinking also suggests a contribution from abnormalities during adolescent brain development. Larson et al. [3] summarized that “*…persons later diagnosed with schizophrenia …*[may show]*… early intellectual and neuromotor disabilities*” [10–14]. By the first episode of psychosis, those affected on average exhibit slightly larger cerebral ventricles and slightly less central gray matter than healthy controls [15]. These findings support the notion that at least part of the disease process appears to be developmental.

CHR for schizophrenia is typically observed in adolescents and young adults who manifest disturbances in stress tolerance, perception, cognitive function, language, motor function, energy, and initiative [16]. Although paranoia and hallucinations may be intermittently reported during the CHR phase, they are recognized by the patient as ‘not real’. The CHR phase ‘ends’ and the diagnosis of psychosis reached when the episodes of paranoia and hallucinations become much more frequent and reality testing wanes. Approximately 20–40 % of subjects with CHR go on to develop psychosis and full-onset schizophrenia [17–22]. Approximately 60–80 % of the subjects with CHR who proceed from prodrome to psychosis can be variably predicted based upon severity of pre-psychotic symptomatology [3].

The ultimate outcome of those patients with CHR symptoms who do not ‘convert’ is poorly established; most continue to exhibit an array of cognitive and behavioral issues. Some are later diagnosed with schizotypal disorder, in the absence of psychosis. Some investigators have questioned whether subjects with CHR symptoms, who do not convert to schizophrenia, should constitute a separate syndrome [23]. Treatment of CHR with atypical antipsychotics, antidepressants, cognitive therapy, or fish oils may result in behavioral improvement; however, initial favorable response to these treatments may not result in long term benefits following treatment cessation [3, 24–29]. A more recent study suggests that fish oils may be of preventative value [30]. The rate of non-conversion, the difficulties prospectively identifying the converters, and the risks of available treatments and stigmatization has made preventive treatment complex.

MRI studies of CHR have variably documented findings of reduction in frontal and temporal grey matter volumes with suggestion that such imaging data could help determine the risk of psychotic manifestations [15, 31–33]. However, an important study by Owens et al. [34] served to diminish these expectations. These authors contrasted MRI-acquired measures of prefrontal cortex grey matter volume reductions and of neuropsychological measures of mental executive function in close relatives of patients with schizophrenia. MRI prefrontal abnormalities were not shown to be familial whereas the neuropsychological findings were familial. The authors concluded “*…that the well-recognized prefrontal volume reductions …*[in schizophrenia]*… are not related to the same familial influences that increase schizophrenia liability and, instead, may be attributable to illness related biological changes or indeed confounded by illness trajectory, chronicity, medication or substance abuse, or in fact a combination of some or all…*” [34].

As has been summarized [35], it remains generally agreed that electroencephalogram (EEG) evaluation by traditional, unaided visual inspection bears scant clinical or scientific fruit for the study of psychiatric patients – aside from its value in excluding epilepsy. This contrasts with an abundance of published evidence that numerical quantification of EEG by spectral and related spectral coherence analyses have demonstrated “*…high proportions of abnormal findings… with good concordance and high specificity… across numerous studies…* [in psychiatric disorders]” [36]. Spectral analysis refers to the quantitative analysis of EEG frequency across spectral bands (e.g. delta, theta, alpha, beta, and gamma) typically by Fast Fourier Transform (FFT) [37, 38]. Spectral coherence refers to the assessment, on a frequency by frequency basis, of the phase difference between the signals from two EEG channels as compared over time. As Duffy and Als stated [35], “*Spectral coherence is a measure of synchronization between two…* [EEG]*… signals based mainly on phase consistency; that is, two signals may have different phases…* [differing relative temporal shifts]*… but high coherence occurs when this phase difference tends to remain constant over time*” [39]. High coherence values are taken as a measure of strong connectivity or coupling between the brain regions that produce the compared EEG signals [37].

Early studies of EEG spectral background in schizophrenia demonstrated excessive frontal delta slowing [40, 41]; however, a later study demonstrated that a major component of the frontal slowing could be attributed to residual frontal eye blink delta band artifact that may survive visually-based attempts at blink removal. Eye blinking has been documented to be more prominent in subjects with schizophrenia [42]. Furthermore, increased spontaneous eye blink frequency has also been well-documented in schizophrenia [43].

Many studies have subsequently evaluated EEG spectral coherence in schizophrenia [44–50]; overall results suggested an increase of both inter- and intra-hemispheric coherence. Indeed, Mann et al. [46] concluded, on the basis of their group data analyses, that “*Increased coherence might be assumed to be a vulnerability marker for schizophrenia reflecting maldevelopment of the brain before onset of the disorder.*” However, during hallucinations, working memory tests and photic stimulation, others have found that coherence may be relatively reduced in schizophrenia [44, 48, 50].

Recently, there has been a focus on the gamma EEG spectral band in schizophrenia [51–60]. This interest arises as cortical gamma oscillation appears to be involved in both local and large-scale neuronal synchronization underlying a number of perceptual and higher order cognitive functions of the sort often found to be abnormal in schizophrenia [51]. Unfortunately, the background EEG gamma spectral band and the spectral band of ambient waking scalp muscle activity, as evidenced in scalp EEG recordings, almost exactly overlap [61]. It has further been observed that ‘thinking’ activates scalp muscle artifact [62]. In addition, the induced gamma-band EEG response (iGBR) recorded on the scalp in response to external stimuli, such as in evoked potential studies, is widely assumed to reflect synchronous neural oscillation associated with object representation, attention, memory, and consciousness. However, it has been suggested that the visual iGBR recorded within scalp EEG may actually reflect properties of visually induced miniature saccade dynamics rather than neuronal oscillations [63, 64], although this has been contested [65]. More recently, it has been reported that auditory iGBR recorded on the scalp may also be affected by saccadic muscle activity [66]. Hence, until signal processing reaches the point where scalp muscle-generated gamma artifact and true scalp recorded cortical gamma activity contributions can be differentiated cleanly and reliably within traditional EEG recordings, scalp frequencies above 30 Hz (gamma) must be considered to be unreliable for the purposes of spectral and coherence analyses, since gamma spectral data appear to be seriously contaminated by background and/or induced muscle activity. Clinical differences of scalp EEG activity between groups in a comparison study of schizophrenic patients and neurotypical control (CON) subjects could result in spurious, albeit statistically significant, high frequency gamma band spectral and/or coherence between groups. To quote Whitham et al. [62] *“…severe restrictions exist on utilizing scalp recordings for high frequency EEG*”.

The intent of the current study was to search for consistent scalp EEG differences (by quantitative spectral and spectral coherence analyses) between patients with CHR for schizophrenia and CON subjects. The goal was to determine (1) whether ambient, waking state EEG recordings provide data which allow reliable group separation; (2) whether the variables best separating the groups contain important information regarding the physiological nature of group difference; and (3) whether a composite, multivariate ‘discriminant function’ might be developed to serve as a potential biomarker [67] for CHR schizophrenia. In addition to gathering and analyzing resting EEG, a steady-state evoked potential, the frequency modulated evoked response (FMAER), was explored. The FMAER arises from the superior temporal (STG), bilaterally, and its use may facilitate physiological investigation of receptive language processing. The FMAER has shown abnormalities in children with a history of sudden-onset regressive autism [68, 69]. The STG is a site of interest having recently been suggested, by Fulham et al. [70], as a marker of abnormal functioning by ‘mismatch negativity’ in schizophrenia and prodrome, and by Oertel et al. [71, 72] as sources of auditory dysfunction in schizophrenia.

To obviate contamination by eye and or muscle artifact in the current study, a multi-step methodological approach was employed that was successfully used by the first author in the evaluation of group differences between children within the autism spectrum and CON subjects [35]. Furthermore, the full EEG analytic approach also relies upon the computational reduction into a manageable number of factors of the large number of coherence and spectral variables produced by single subjects. This computational process facilitates an objective approach to data management for subsequent group analyses [73, 74]. It obviates the need for *a priori* preselection of a subset of coherence variables in order to avoid Type 1 and 2 statistical errors [74, 75].

## Methods

### Venue

All neurophysiological data collection and analysis was performed under the direction and supervision of the first author at the Developmental Neurophysiology Laboratory, Department of Neurology, Boston Children’s Hospital (BCH), a university-affiliated (Harvard Medical School) academic medical center.

### Subjects

#### Patients with Schizophrenia Prodrome Syndrome (CHR)

The CHR screening assessment included administration of the Schedule for Affective Disorders and Schizophrenia for School-Age Children-Present and Lifetime Version [76]; the K-SADS, as it is known, constitutes a validated semi-structured interview used to diagnose mood, anxiety, substance abuse, and psychotic disorders in youth under the age of 18. Both participant and parent/guardian report of the participant’s symptom history were solicited for the current study.

A number of tests/indices have been devised in order to quantify identification and study of the CHR state [3, 77]. The current study employed the Scale of Prodromal Symptoms (SOPS) [78, 79]. The SOPS is embedded within an interview form, the Structured Interview for Prodromal Syndromes (SIPS), designed to diagnose prodromal (CHR) syndromes according to published criteria and to rate severity of CHR symptoms [19]. While the SIPS serves to define/identify the CHR state it does not in itself serve to identify later development of psychosis. The SIPS and the SOPS [80] were administered to all participants as part of the screening.

SIPS/SOPS raters were certified through a standard 1½-day training program developed by the assessment’s creators at Yale University’s PRIME Research Clinic. At the start of the current study, raters also attended the Boston site for the North American Prodromal Longitudinal Study (NAPLS-2) to study SIPS interview collection and scoring for 9 months to ensure consistent ratings across sites. Among the current study sample, 66 (97 %) of the SIPS/SOPS were performed by BCH staff; SIPS/SOPS scores for the remaining two participants were provided by their referral source, one from the NAPLS-2 and one from the Social Neuroscience and Psychopathology Laboratory of Harvard University, as they had been assessed at these laboratories within 30 days of entering the current study.

In addition, information about past medical history, medication usage, school functioning, and academic functioning was obtained. The participant’s parent/guardian and treating clinician were questioned to determine that the participant was functioning at grade level in a regular classroom without special education services and was without any other evidence of intellectual and/or academic disability. To further screen for academic and intellectual function outside normal range, the Scales of Independent Behavior-Revised was performed; this comprehensive scale constitutes a norm-referenced assessment of adaptive and maladaptive behaviors [81]. Only participants with positive SIPS/SOS scores but without evidence of academic or intellectual disability were included in the current study.

On the day of the laboratory visit, the participant’s parent/guardian completed a demographic questionnaire that asked for report of the participant’s demographic information and medication usage. Parents/guardians were asked to provide consent for review of medical records to further characterize participants’ mental health history. If more than 1 month had elapsed since the screening assessment, participants were re-administered the SIPS/SOPS to confirm that the participant remained within their previously determined clinical group. No participant was reclassified based upon reassessment.

Study subjects were recruited, under the direction of the senior author, from among three sources: clinical referrals to the BCH outpatient psychiatric clinic, the NAPLS-2 program, and the Social Neuroscience and Psychopathology Laboratory. Inclusion criteria were (1) a clinical diagnosis of ‘schizophrenia prodromal (CHR) syndrome’, including documentation by a senior staff psychiatrist of the patient’s report of intermittent cognitive distortions (e.g. hallucinations, paranoia, delusions); (2) positive SOPS scores from the SIPS as administered by trained and certified technologists and confirmed by a staff psychiatrist; and (3) written agreement as required by the BCH Institutional Review Board (IRB) of the patient and/or parent/guardian to participate in the study. Exclusion criteria were the presence of any of the following: (1) co-existing primary neurologic syndromes (e.g. Trisomy X or Klienfelter’s syndromes, tuberous sclerosis, traumatic brain injury, global developmental delay, developmental dysphasia, hydrocephalus, hemiparesis, or any other known syndromes affecting brain development); (2) coexisting primary psychiatric syndromes (e.g. depression, bipolar disorder, attention deficit hyperactivity disorder, obsessive-compulsive disorder); (3) clinical seizure disorders or results of prior EEG readings suggestive of an active seizure disorder or epileptic encephalopathy; (4) report of major medical illnesses (e.g. diabetes, severe asthma, cardiovascular abnormality, endocrine abnormality, etc.); (5) taking prescription medication(s) at the time of study; or (6) significant primary sensory disorders (e.g. blindness and/or deafness).

#### Healthy, CON subjects

Healthy CON subjects were recruited by poster and colleague/acquaintance referral. None were recruited from within families of the subjects with schizophrenia prodrome syndrome. Controls were screened by the same procedure as described above for the CHR subjects. Inclusion criteria required (1) the absence of any symptoms of schizophrenia and (2) signed IRB consent as indicated above. Exclusion criteria were the presence of any of the following: (1) any neurological, psychiatric, and/or medical illnesses and/or primary sensory disorders, as for the CHR (prodrome) group spelled out above; (2) family history of schizophrenia or other major mental illness; (3) history of drug abuse; (4) non-specific ‘suspicious’ affect, appearance, or behavior as observed by the study personnel; or (5) taking prescription medication(s) at the time of study.

#### IRB approval

All subjects and/or their families, as age appropriate, gave written informed consent in accordance with protocols approved by the IRB of the BCH Office of Clinical Investigation. The approved protocol is in full compliance with the Helsinki declaration.

### Data acquisition

#### Neurophysiology recording: EEG data collection and initial processing

All subjects’ electrophysiological data obtained for this study were gathered by technologists trained and supervised by the first author, an experienced academic clinical electroencephalographer. Data were collected with an EGI™ 128 channel geodesic net system (Electrical Geodesics Inc., Eugene, OR, USA) along with a single information channel dedicated to a stimulus trial marker utilized for evoked potential collections (see FMAER below). A conductive gel rather than a salt solution was employed with the electrodes. Disadvantages of salt-soaked sponge electrode use include inter-electrode conductive ‘salt bridges’ and high electrode-scalp impedance due to more rapid drying out, both of which may lead to a difficult-to-detect increase in artifact. All subjects were studied in a sound and electronically (Faraday) shielded chamber and were visible and easily accessible to the technologist via one-way mirror window and door. The recording equipment stood outside of and immediately adjacent to the recording chamber. Data were sampled at 500 Hz with 0.1–100 Hz EEG band pass. Several separate epochs of eyes closed, waking state data were obtained over the course of the study with frequent breaks and verbal interchange to facilitate alertness. Approximately a total of 20 minutes of apparently artifact-free waking EEG was recorded per subject. Eyes closed, waking state ambient EEG recordings were temporally interdigitated with sessions of evoked potential recordings (see below, FMAER). Frequent rest breaks were built in as indicated. After conclusion of data collection, all research subjects with electrode nets in place underwent photogrammetry with an 11 camera-based EGI system, so as to establish the precise position of the 128 net electrodes and thus to facilitate off-line mapping to standard EEG electrode positions for comparative purposes. Data were then de-artifacted (see below, Artifact management – part 1 and part 2), re-montaged by 3D spline interpolation, and signal averaged as indicated (FMAER) by BESA™ software (BESA GmbH, Gräfelfing, Germany). Original unprocessed data were permanently archived within a Developmental Neurophysiology Laboratory database.

**Fig. 1:**
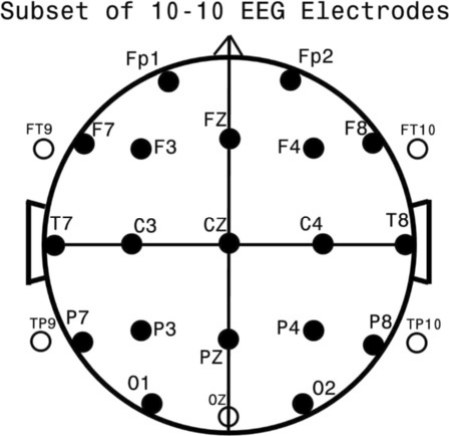
Standard EEG electrode names and positions. Head in vertex view, nose above, left ear to left. EEG electrodes: Z, Midline; FZ, Midline frontal; CZ, Midline central; PZ, Midline parietal; OZ, Midline occipital. Even numbers, right hemisphere locations; odd numbers, left hemisphere locations: Fp, Frontopolar; F, Frontal; C, Central; T, Temporal; P, Parietal; O, Occipital. The standard 19, 10–20 electrodes are shown as black circles. An additional subset of five, 10–10 electrodes are shown as open circles. This figure is reproduced from a prior publication [35] with permission

For subsequent analysis, EEG, spectral, coherence, and FMAER data were additionally reduced in number by BESA using 3D spline interpolation to 24 standard EEG locations (FP1, FP2, F7, F3, FZ, F4, F8, T7, C3, CZ, C4, T8, P7, P3, PZ, P4, P8, O1, OZ, O2, FT9, FT10, TP9, TP10 – see Fig. 1 for standard EEG electrode placement), bandpass filtered from 0.5–50 Hz, mains filtered at 60 Hz, and down-sampled to 256 Hz so as to reduce data dimensionality and facilitate comparison to data gathered with more common lower temporal and spatial resolution recording systems.

#### Neurophysiology recording: the Frequency Modulated Auditory Evoked Response (FMAER)

The FMAER stimulus was initially derived by Green and Stefanatos [82–86] as a means to assess the temporal lobes’ ability to decode rapidly changing speech patterns, essential to the accurate detection of phonemes and, in turn, essential for language decoding and ultimately language comprehension. By means of source analysis the FMAER has been shown in neurotypical subjects to arise from both STG [68]. The FMAER is formed by starting with a 1000 Hz sine wave and frequency, modulating it with another 10 Hz sine wave, which results in ‘warbling’, and then further modulating it by slowly turning the warbling on and off with another 4 Hz sine wave [68]. A trial marker locked to the onset of one second of the 4 Hz sine wave and saved with concurrently recorded EEG produces, after signal averaging, a scalp recorded 4 Hz sine wave in normal subjects. The FMAER stimulus and trial marker are created by a stand-alone Spark2 generator (Mind Spark Inc., Newton, MA, USA). The auditory signal is bi-aurally presented by speakers at 78 db sound pressure level.

The FMAER response may be absent in the Landau-Kleffner syndrome when language deteriorates [64] and in autistic children with histories of rapid language and/or behavioral regression [65]. Successful pharmacologic treatment may improve receptive and expressive language function and restore a previously absent FMAER [64, 65]. FMAER spectral analysis has also shown that the FMAER scalp response may be as much ‘distorted’ as ‘absent’ in regressive autism associated with language loss [65].

#### Measurement issues and solutions: artifact management – part 1

After each subject’s participation in the EEG study, EEG epochs were inspected by the EEG technologist to visually identify which epochs were recorded during breaks for relaxation, or showed movement artifact, electrode artifact, eye blink storms, drowsiness, epileptiform discharges, and/or bursts of muscle activity. When so identified, they were marked for exclusion from all subsequent analyses. The EEG technologist’s results were reviewed for accuracy by the first author, who then removed remaining eye blink and eye movement artifacts, which may be surprisingly prominent even during the eyes closed state, by utilization of the source component technique [87–89] as implemented in BESA software. These combined techniques resulted in EEG data that appeared largely artifact free, with rare exceptions of low level temporal muscle artifact and persisting low voltage frontal and anterior temporal slow eye movement, which however may contaminate subsequent analyses. The final reduction of any persisting contamination of processed variables (coherence) is discussed below under Artifact management – part 2.

### Data processing

#### Calculation of spectral coherence and spectral variables

As previously described [35], 8–20 minutes of eyes closed, awake state EEG cycles per subject were transformed within BESA to the Laplacian or current source density reference. This approach provided reference-independent data that are primarily sensitive to underlying cortex and relatively insensitive to deep/remote EEG sources. Use of current source density reduces spurious effects of volume conduction upon coherence by emphasizing sources at small spatial scales [90].

Spectral coherence was calculated using a Nicolet™ (Nicolet Biomedical Inc., Madison, WI, USA) software package, according to the conventions recommended by van Drongelen [37, p. 143–4, equations 8.40, 8.44]. In practice, coherence is typically estimated by averaging over several epochs or frequency bands [37]. In the current project, a series of 2 second epochs was utilized to process available EEG segments. Spectral coherence measures were derived from the 1–32 Hz range, in 16 2-Hz-wide spectral bands resulting in 4,416 unique coherence variables. The 24 × 24 electrode coherence matrix yields coherence values where the matrix diagonal has a value of 1 – each electrode to itself – and half of the 552 remaining values duplicate the other half. This results in 276 unique coherences per spectral band. Multiplication by the 16 spectral bands in turn results in 4,416 unique spectral coherence values per subject [35].

Standard spectral data were calculated using the common average reference by FFT over the same frequency range noted above and based upon the FFT algorithm described in Press et al. [38, p. 411–2]. Resulting spectral data were utilized in order to approximate residual artifact contamination (see Artifact management – part 2) and as potential predictor variables. Per subject, the 24 EEG channels and 64 spectral bands per channel result in 1,536 spectral data values.

#### Measurement issues and solutions: artifact management – part 2

As previously detailed [35], visual inspection or direct elimination of electrodes and/or frequencies where a particular artifact is most easily apparent do not remove all artifact from an EEG data set on their own. An established approach to further reduce any persisting artifact contamination of processed coherence data involves multivariate regression. Semlitsch et al. [91] demonstrated that, after identifying a signal that is proportional to a known source of artifact, this signal’s contribution to scalp recorded data may be diminished by statistical regression procedures. As also previously detailed [35], persisting vertical eye movements and blinks produce slow EEG delta spectral signals in the frontopolar channels FP1 and FP2. Such artifact contribution may be estimated by the average of the 0.5 and 1.0 Hz spectral components from these channels after EEG spectral analysis by FFT of common average referenced data. Similarly, horizontal eye movements may be estimated by the average of the 0.5–1.0 Hz spectral components from anterior temporal electrodes F7 and F8. Little meaningful EEG information of brain origin is typically found at this slow frequency in these channels in the absence of extreme pathology. Muscle activity tends to peak at frequencies above those of current interest. Accordingly, 30–32 Hz spectral components were considered to be largely representative of muscle contamination, especially as recorded from the separate averages of prefrontal (FP1, FP2), anterior temporal (F7, F8), mid-temporal (T7, T8), and posterior temporal (P7, P8) electrodes. These electrodes are most often contaminated by muscle artifact as they are physically closest to the source of the artifact, namely the frontal and temporal muscles. The steps employed in the current study involved, first, the fitting of a linear regression model where the dependent variables were those targeted for artifact reduction and the independent variables were those chosen as representative of remaining artifacts; second, the extraction of the residuals, which now represented the targeted data after artifact removal; and, third, the use of the residuals in subsequent analyses. The six artifact measures, two very slow delta and four high frequency beta measures, were submitted as independent variables to a multiple regression analysis (BMDP2007™-6R) [92] in order to individually predict each of the coherence variables (see below), which were treated as the dependent variables. The residuals of the dependent variables, now uncorrelated with the chosen independent artifact variables, were used in the subsequent analyses. The above regressions were performed separately on both spectral and coherence data sets prior to principal components analysis (PCA; see below).

#### Prevention of capitalization upon chance: variable number reduction by creation of spectral and spectral coherence factors

Spectral and spectral coherence analyses produce many variables per subject. Steps must be taken to avoid capitalization on chance, which may result from the use of too many variables. Typically, the number of variables is reduced based upon expectations from results of prior analyses and/or current hypotheses. A more objective approach follows the advice of Bartels [73, 93], who proposed establishment of the intrinsic data structure within large data sets by use of PCA, and utilization of the resulting smaller set of computed factors to represent the subjects in subsequent analyses. Modern texts continue to recommend PCA for variable reduction [94, 95]. Spectral and spectral coherence data were first normalized (centered and shifted to have unit variance) so that eventual factors reflect deviations from the average. The PCA-generated smaller set of factors that represents a large portion of the original variance results in a substantial reduction of the ultimate variable number per subject. This obviates the need for the ‘expert guided’ selection of variable subsets for subsequent statistical analyses with resulting risk of type 1 and type 2 statistical error. A data set of uncorrelated (orthogonal) factors is produced in which a small number of orthogonal factors are identified following varimax rotation.

**Fig. 2:**
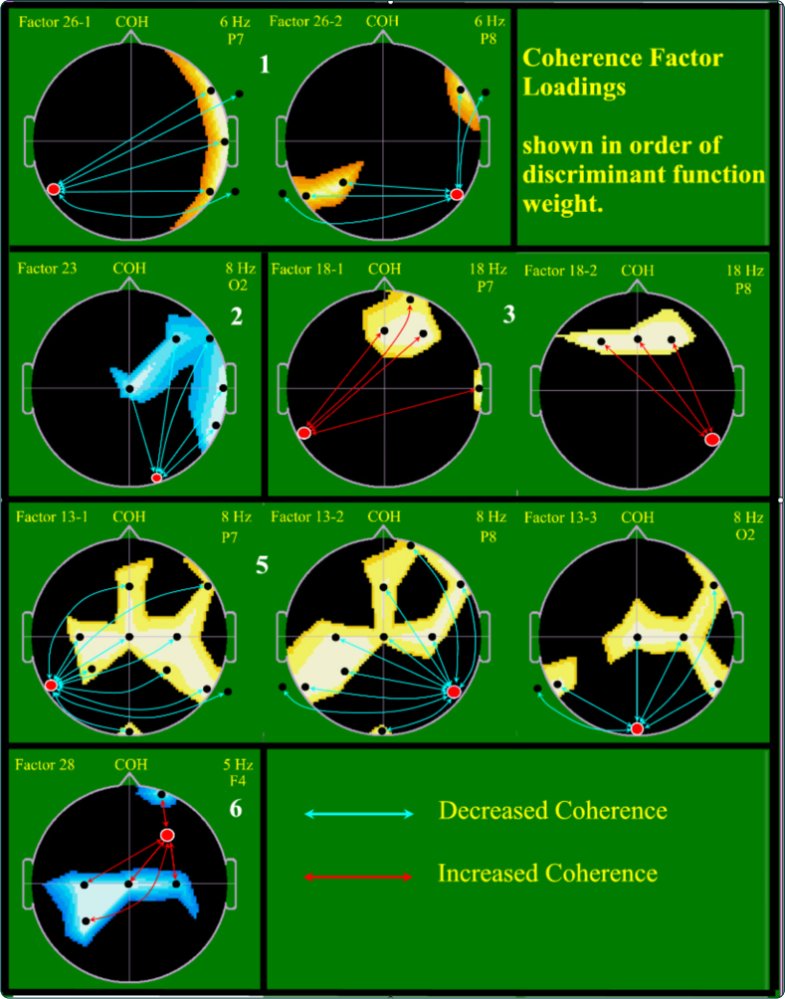
Factor loadings for five coherence factors chosen to best differentiate clinically high risk (CHR) from neurotypical controls. Schematic heads are shown in vertex view, scalp left to image left with nose above. Each one of the five black-bordered rectangles or squares displays, within its borders, information relevant to a single one of the five coherence factors selected by discriminant function analysis (DFA; see text). For example, the first row displays data describing the first factor chosen by DFA, Factor 26. Factor name is shown above and to the left of each image (e.g. Factor 28), in yellow. Above the nose ‘COH’ indicates that the image displayed is a coherence factor. Where a factor requires more than one image to illustrate relevant coherence loadings, they are separately labeled (e.g. Factor 26–1, Factor 26–2). The order of selection by DFA for each coherence factor within the overall choice of eight factors is shown as a large white number. To the top right of each image is the relevant spectral frequency and primary index electrode, displayed in yellow (e.g. 6 Hz P7). The colored regions within the images reflect the region and sign of coherence loadings from the initial PCA. The index electrode for each image is show as a red circle bordered in white. Lines connect this index electrode to additional electrodes (black dots). Line color reflects reduced (blue) or increased (red) coherence for the CHR population

**Fig. 3:**
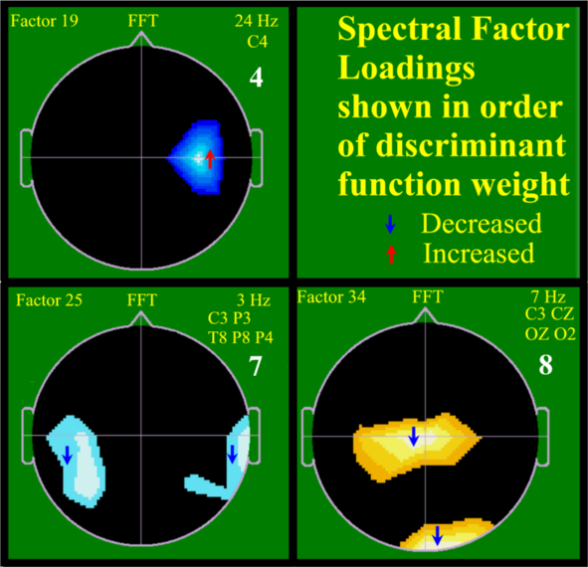
Factor loadings for three spectral factors chosen to best differentiate clinically high risk (CHR) from neurotypical controls. Schematic heads are shown as for Fig. 2. Each of the three black-bordered squares displays information relevant to one of the three spectral factors selected by discriminant function analysis (DFA; see text). Above the nose “FFT” signifies the image displayed is a spectral factor. Relevant spectral bands and electrodes are shown above and to the right. The order of selection by DFA for each spectral factor within the overall choice of eight factors is shown as a large white number. For example, the first square displays data for FFT Factor 19 involving 24-Hz activity at electrode C4 and was the fourth factor chosen by DFA. The colored regions within the images reflect the region and sign of spectral loadings from the initial PCA. Color of a small associated arrow reflects reduced (blue) or increased (red) spectral activity for the CHR population

Each factor is formed as linear combination of all input variables with the weight or loading of each coherence variable upon a particular factor as determined by the PCA computation [73]. Meaning of outcome factors is discerned by inspection of the loadings of the input variables upon each individual factor [73, 96]. Factor loadings were treated as if they were primary neurophysiologic data and displayed topographically [97, 98]. A display of approximately the highest 15 % of coherence loading values was utilized to facilitate an understanding of individual factors’ meaning (Figs. 2 and 3). This approach has been used successfully for both spectral [99] and spectral coherence [35, 100–102] data reduction and analysis.

#### FMAER: spectral signal and noise analysis

As described above, a normal subject’s 4 Hz FMAER appears, upon scalp recording, in the form of a one second 4 Hz sine wave. Abnormal FMAERs – as observed in some children with Landau-Kleffner syndrome or autism – appear as noisy, distorted, or partial 4 Hz sine wave, or occasionally just as low amplitude noise [68, 69]. ‘Noisy’ responses may reflect four possibilities: (1) there is no 4 Hz response and the noise reflects residua of incomplete signal averaging; (2) a low amplitude 4 Hz response is present but is partially masked by noise from incomplete averaging; (3) the response itself is distorted, causing a non-sinusoidal appearance (side-band noise); or (4) a combination of these possibilities. A study of children with regressive autism using spectral analysis of FMAERs formed from both standard averaging and ‘plus-minus’ averaging [37] demonstrated that, at baseline, children with absent language demonstrated FMAERs manifesting distorted processing, i.e. the 4 Hz auditory input to the ears produced, instead of a clean 4 Hz scalp sinusoid, a broad-band 2–7 Hz response [69]. Accordingly, spectral analysis was performed on the current study subjects’ FMAERs in order to search for evidence of auditory processing distortion in schizophrenia prodrome. FFTs were formed on all subjects in the current populations at 4 Hz (response frequency) as well at 3 Hz and 5 Hz (sideband noise frequencies).

### Data analysis

#### Discrimination of subject groups by use of EEG spectral coherence and spectral variables

Two-group DFA [96, 103, 104] was used in the current study. As previously described [35], it produces a new canonical discriminant variable which maximally separates the groups based on a weighted combination of the entered variables. DFA defines the significance of a group separation, summarizes the classification of each subject, and provides approaches to the prospective subjective classification by means of the jackknifing technique [105–107]. The BMDP statistical package [108] was employed for DFA (program 7 M) which yields the Wilks’ Lambda statistic with Rao’s approximation. For the estimation of prospective classification success, the jackknifing technique was used [105–107] as provided within program 7 M. In jackknifing for two-group DFA, the discriminant function is formed on all subjects but one. The left-out subject is subsequently classified. This initial left-out subject is then folded back into the group (hence “jackknifing”), another subject is left out, the DFA is performed again, and the newly left-out subject classified. This process is repeated until each individual subject has been left out and classified on the basis of the ‘non-left-out’ subjects. The assessment of prospective classification success is based upon a tally of the left out subjects’ correct classification. This technique is also referred to as the ‘leaving-one-out’ process and is generally taken as an estimate of future classification success for populations of the size used in this project. A better estimation of classification success involves multiple split-half replications as previously demonstrated on a large population of autistic children [35]. It is notable that, within the autism study the group, the average split half classification success and the corresponding jackknifed classification were quite comparable.

## Results

### Subjects

Over the past 4 years, 35 CON subjects were recruited and 57 putative patients with CHR were identified. Twenty of the 57 manifested psychosis and an additional 15, although non-psychotic, had been placed on medications by the time of neurophysiologic evaluation. Accordingly, these 35 were excluded from the study target sample of unmedicated patients with CHR. The remaining 22 non-psychotic, unmedicated CHR patients made up the population to be contrasted with the CON group. The final data analyses were, therefore, based upon 35 control subjects and 22 subjects with unmedicated CHR. Relevant, selected CON and CHR group demographics are shown in Table 1.

**Table 1:** Control and high risk population demographics

Variable	Controls	Behavioral high risk	Fisher exact test
	n = 35	n = 22	
Sex	14 males	11 males	n/s
	21 females	11 females	
Handedness	34 right	20 right	n/s
	1 left	2 left	
		Mean ± SD	
Age at study	19.175 ± 5.690	13.679 ± 3.240	T-test = 4.04; *P* ≤0.0002

n, group population size; n/s, not statistically significant; *P*, probability

EEG data were collected on the full population of 92 subjects and all were utilized for the purpose of coherence and spectral variable reduction by PCA so as to maximize the information/variance content within the PCA analyzed data set.

There were no significant differences between the CON and CHR groups in terms of handedness and sex. Significant difference in mean age between these two groups was managed by removing the effect of age upon variables, prior to use in discriminant analysis, using statistical regression.

### Factor development by PCA

Data reduction (factor formation) was independently performed for the 4,416 spectral coherence variables and the 1,536 spectral variables. For PCA on the coherence data, results demonstrated, after varimax rotation, a good condensation of variance upon a small number of factors. After varimax rotation, the first coherence factor accounted for 2.98 % of the total variance contained within the full set of the original 4,416 coherence variables. Five factors accounted for 15.14 % of the original variance, and the full 40 factors accounted for 77.82 % of the overall variance. For PCA on the spectral data, results demonstrated even stronger data condensation. After varimax rotation the first spectral factor accounted for 14.13 % of the total original variance, five factors accounted for 44.07 % of the variance, and the full 40 factors accounted for 95.92 % of the total, original variance. Age at study was regressed (BMDP-6R) from all factors before use in subsequent analyses. Loadings of original coherence and spectral variables upon corresponding summary factors are illustrated in Figs. 2 and 3.

### Discriminant function analysis (DFA)

**Fig. 4:**
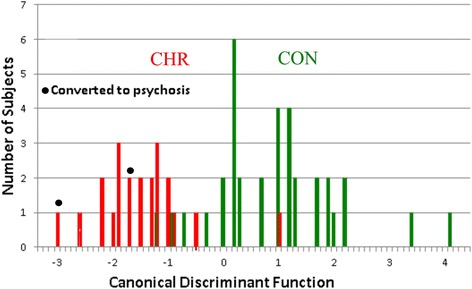
Clinical high risk (CHR) and neurotypical control (CON) population distributions on the discriminant function analysis-derived canonical discriminant variable. Population distributions are shown for the CON (green) and CHR (red) groups. The X axis is the canonical discriminant, ranging from +4.5 to −3.5 units, which was created by the DFA process utilizing the eight factors described in Table 2, part (3). The Y axis represents subject number

Two-group stepwise discriminant function analysis (BMDP 7 M) was instituted contrasting groups CON (n = 35) and CHR (n = 22), where coherence and spectral factors were allowed to enter (F to enter 4.0, to remove 3.996). Results are shown in Table 2. As evident, the F statistic approximation to Wilks’ Lambda was statistically significant (*P* ≤0.00001). Five coherence and three spectral factors were utilized (Table 3). The first three variables chosen and five of the eight chosen variables were coherence factors. Initial classification was 91.2 % correct overall (CON, 88.6 %; CHR, 95.5 % correct). The classification success upon completion of jackknifing was 86.0 % correct overall (CON, 85.7 %; CHR, 86.4 % correct). The groups where displayed as a histogram on the canonical discriminant variable (generated from the eight chosen factors) showing a mostly bimodal distribution (Fig. 4).

**Table 2:** Discriminant function analysis controls (CON) vs. clinical high risk (CHR)

(1) Standard analysis			
Group	Percent	Number of subjects classified	
	Correct	CON	CHR
CON	88.6	31	4
CHR	95.5	1	21
TOTAL	91.2		
(2) Jackknife analysis			
Group	Percent	Number of subjects classified	
	Correct	CON	CHR
CON	85.7	30	5
CHR	86.4	3	19
TOTAL	86.0		
(3) Eight factors chosen to make discrimination, in order of choice			
		Overall estimated significance	
	1. COH 26	Wilk’s lambda = 0.419	
	2. COH 23	DF = 8, 48	F = 8.313 p = 0.00001
	3. COH 18		
	4. FFT 19		
	5. COH 13		
	6. COH 28		
	7. FFT 25		
	8. FFT 34		

COH, Coherence factor; FFT, Spectral factor; DF, Degrees of freedom for F test; F, F statistic; *P*, Estimated probability

**Table 3:** Sample frequency modulated auditory evoked response (FMAER) scores

	Mean ± SD		
Variable	Controls	High risk	Probability
3 Hz-TP9	0.0331 ± 0.102	0.0301 ± 0.055	n/s
4 Hz-TP9	0.6892 ± 1.707	0.5471 ± 0.584	n/s
5 Hz-TP9	0.0456 ± 0.014	0.1441 ± 0.039	n/s
3 Hz-TP10	0.0022 ± 0.055	0.0407 ± 0.058	n/s
4 Hz-TP10	0.8177 ± 0.531	0.9479 ± 0.708	n/s
5 Hz-TP10	0.0539 ± 0.008	0.1231 ± 0.027	n/s

4 Hz, Targeted signal frequency; 3 Hz, 5 Hz, Side band frequencies; TP9, TP10, Respective left and right posterior temporal regions, expected sites of maximum left and right sided FMAER response (typical FMAER waveforms, normal and abnormal, may be viewed in on-line references [64, 65]); n/s, Not significant by Student’s t-test

### Factor loadings

Figure 1 illustrates the standard placement for the 24 EEG electrode locations utilized, whereas Figs. 2 and 3 illustrate the loading patterns for the five chosen coherence factors and for the three chosen spectral factors, respectively, as utilized in the above DFA. Three of the five coherence factors involved numerous coherence loadings and, for the purpose of clarity, more than one image was utilized for demonstration, e.g. the first coherence Factor 13 is depicted in three separate images. Each image is shown in vertex view, i.e. from above; the left ear is shown to the left and the nose above.

In each coherence factor image the index electrode and primary EEG frequency are named top right and the referenced electrode is also shown in red with a white circle. Blue or red lines with arrow tips indicate the other involved electrode (black circle) for each involved coherence pair. Blue lines indicate reduced coherence for the CHR group and red lines indicate increased coherence for the CHR group in the featured DFA. Three coherence factors demonstrated reduced coherence (Fac 26, 23, 13) and two increased coherence (Fac 18, 28). Note that the first two chosen factors manifest reduced coherence. Thus, although both reduced and increased coherence are observed, reduced coherence predominates. Also note, in Fig. 2, that long distance coherence (coherence other than to adjacent electrode) predominates. No single coherence factor demonstrated a combination of decreased and increased factor loadings. Four coherence factors involved both hemispheres (Fac 26, 18, 13, 28) and one factor just involved the right hemisphere (Fac 23). Involved spectral bands were limited to the theta (Fac 26, 23, 13, 28) and slow beta (Fac 18) spectral bands.

The colored background in the illustrated coherence factors indicates the coherence loading from the original PCA which, in combination with the loading of the PCA-produced factors on the discriminant canonical variable, determines the color of the lines (red or blue, see below). Regions involved by coherence factor index electrodes (red, white circle) are temporal – six (within Fac 26, 18, 13), occipital – two (Fac 23, 13), and frontal – one (Fac 28). Of the six temporal index electrodes, all involved the posterior temporal regions, three left and three right sided. All six coherence factors involved primarily long distance associations. None involved predominantly short distance associations.

For each of the three spectral (FFT) factors used in the DFA, regions involved are illustrated in color, which reflects loading of the regions’ spectral content upon the given factor in the PCA. Next to each region is a small arrow, the color and direction of which reflects spectral change for the CHR group in the DFA (red, up, increase for CHR group; blue, down, reduction in CHR group). Just one factor demonstrated increased CHR spectral magnitude (Fac 19) and two manifested decreased magnitude (Fac 25, 34). All spectral factors reflected one single spectral band: delta (Fac 25), theta (Fac 34), and beta (Fac 19). Areas involved by the spectral factors included the central-parietal (Fac 4, 25, 34), temporal (Fac 25), and occipital (Fac 34) regions. None of these regions are typically associated with eye or muscle artifact.

### FMAER data analysis

The FMAER paradigm was performed on the 35 CON and 22 CHR subjects. A standard average was created as well as a plus-minus average. The former creates an average combination of the signal response, average of noise within the response, and residual background noise left after averaging. The latter produces an estimate of the residual background noise left after averaging [37, p. 61]. Both the standard and the plus-minus average response were spectral analyzed by BESA from 3 through 5 Hz across all 24 channels utilizing the common average reference. Subtraction of spectral analysis of the plus-minus average from that of the standard average created a spectral difference plot where random background noise is removed but the response remains. Ideally, the 3 Hz and 5 Hz sideband spectral values should be zero and the 4 Hz component should be prominent. When the subtracted values for the groups were compared by Student’s t-test, no significant differences were observed for any frequency or electrode. Table 1 shows the results for the bilateral posterior-inferior temporal electrodes TP9 and TP10 (Fig. 1), electrodes that typically reflect maximal FMAER scalp amplitude [64, 65]. Thus, there was no FMAER evidence for any difference in response (at 4 Hz) magnitude or response distortion (at 3 or 5 Hz) between the CHR and the CON group at the two electrodes typically showing greatest response amplitude [68, 69]. Note the large difference in mean magnitude between the 4 Hz signal and the adjacent 3 and 5 Hz sideband noise components for both studied groups. In other words, both groups manifested normally appearing FMAER responses.

Table 4 illustrates the expected large SOPS scores differences between the CON and CHR groups.

**Table 4:** Structured Interview for Prodromal Syndromes (SIPS) – Scale of Prodromal Symptoms (SOPS) scores

	Mean ± SD			
Variable	Controls	High risk	T-test	Probability
P_MAX_	0.486 ± 0.781	4.333 ± 0.658	19.720	0.0000
P_AVG_	0.177 ± 0.177	2.429 ± 0.985	10.020	0.0000
N_MAX_	0.114 ± 0.323	1.875 ± 0.409	7.680	0.0000
N_AVG_	0.033 ± 0.017	1.395 ± 0.304	5.880	0.0000
D_MAX_	0.200 ± 0.531	2.810 ± 0.273	9.090	0.0000
D_AVG_	0.071 ± 0.215	1.202 ± 0.835	6.090	0.0000
G_MAX_	0.171 ± 0.514	3.381 ± 1.600	8.940	0.0000
G_AVG_	0.057 ± 0.172	2.131 ± 1.264	7.480	0.0000

P, positive symptoms; N, negative symptoms; D, disorganized symptoms; G, general symptoms; MAX, maximum of six scale scores per symptom type, per subject; AVG, average of six scale scores per symptom type, per subject

## Discussion

The objectives of the present study included the development of neurophysiological descriptors of schizophrenia prodrome (CHR) derived from ambient resting EEG. The descriptors were expected to fulfill a series of criteria, included (1) freedom from artifact contamination; (2) being objective and bias free; (3) useful in the quantitative classification/identification of schizophrenia prodrome (CHR); (4) useful in creation of putative, quantitative biomarker for CHR; (5) clinically interpretable; and (6) facilitating assessment of the functional integrity of the STG.

First, although infrequently emphasized, artifact may significantly interfere with group study results that involve quantification of EEG. Excessive artifact adds noise, thereby obscuring discovery of significant group differences (type 2 statistical error, false negatives), or presents asymmetrically within one group causing a spurious group difference (type 1 error, false positives). In order to obviate or at least minimize such possibilities, a four-part process was undertaken. Initially, prominent artifact containing EEG segments were removed by expert visual inspection of EEG. Then, eye blink was computationally eliminated by a source analysis technique [87–89]. Next, small residua of eye movement and muscle were removed by a regression technique [91]. Finally, higher frequencies above 30 Hz (gamma band) were excluded from analysis, in agreement with literature suggesting that such gamma activity is strongly dominated by cranial muscle activity [61]. As a result of the above process, factor loading patterns do not suggest artifactual origins. Moreover, the statistical regression assures that utilized variables are ‘artifact free’, i.e. statistically uncorrelated with (orthogonal to) quantitative estimates of artifact sources.

Second, as originally proposed by Bartels [73, 93], and subsequently refined for use with neurophysiological data [74] and utilized in this laboratory [35, 74, 100, 102], PCA constitutes an objective way to demonstrate the fundamental structure within a data set and simultaneously an objective way to reduce variable number without need for a priori intervention. In this study of coherence data, 40 factors represented 78 % of the information (variance) within the 4,416 original spectral coherence variables per subject. For spectral data, 40 factors represented 96 % of the information within the 1,536 variables per subject. This constitutes a substantial, unbiased reduction of data dimensionality for both spectral and coherence data sets. The high retention of variance indicates that, while reducing data dimensionality, the PCA process preserved the majority of information contained within the original data set variables.

Third, stepwise discriminant analysis (DFA) between the CON and prodrome (CHR) groups demonstrated successful group separation with 91 % correct initial subject classification. This was done on the basis of eight variables, yielding a favorable subject to variable ratio of 57/8, approximately 7:1 [109]. More importantly, jackknifing [105, 106] demonstrated 86 % correct classification for both groups, a favorable indicator [103, 110] for the prospective ‘diagnostic utility’ of EEG derived spectral and coherence data.

Fourth, the DFA canonical discriminant function variable graph (Fig. 4) showed a bimodal distribution between the CON and CHR. This raises the possibility that such a composite univariate discriminant variable formed on larger populations might serve to facilitate identification of response to intervention and/or subsequent conversion to schizophrenia. For example, it is speculated that a given CHR intervention appearing to have little or no positive clinical effect might, in fact, move a subject or subjects along the EEG discriminant from the CHR towards the CON region. Perhaps combinations of such apparently clinically silent, but favorably EEG active, interventions might in aggregate prove to be of combined clinical value. To facilitate the above, PCA and DFA analyses performed for this study allow new subjects (or subjects before and after intervention) to be passed through factor formation and discriminant classification.

Fifth, factor loading plots (Figs. 2 and 3) showed coherence patterns determined by the intrinsic data structure and did not manifest the typical, orderly left-right inter-hemispheric and anterior-posterior intra-hemispheric coherence patterns often pre-selected for analysis. Index coherence electrodes were mostly located over temporal lobes, predominantly involving the posterior temporal regions. Next, most prominent were occipital and frontal index electrodes. Although factors representing both decreased and increased coherence were observed, reduced coherence predominated, with long distance coherence patterns prevailing. From the seven DFA-selected spectral factor plots, none involved the prefrontal and only one the temporal region suggesting successful avoidance, or at least minimization, of spurious artifact dominated variables [40–42].

In agreement with many studies suggesting temporal lobe abnormalities in schizophrenia [33, 71, 72, 111–119] and schizophrenia prodrome [33, 112, 113], the current coherence findings strongly implicate the bilateral temporal areas. Reduced posterior temporal connectivity might be relevant in terms of the complex language disorders in schizophrenia [120] that presumably extend as well to CHR [121]. Our coherence findings are also in line with the studies of Oertel-Knöchel et al. [71, 72], who found functional MRI (fMRI) evidence of reduced connectivity with the planum temporale and the STG within schizophrenics and close relatives. These authors speculated that such findings might be associated with psychotic symptoms, especially auditory hallucinations. Moreover, Mou et al. [122] showed that schizophrenics who report auditory verbal hallucinations and who also demonstrated measured deficits in voice identity recognition, manifest impaired frontal-temporal connectivity. The current study’s coherence factors 18, 13, and possibly 28 may be consistent with such altered frontal-temporal connectivity. In addition, the current findings of mixed but predominantly widespread reduced coherence in the awake resting state contradict prior speculations that increased coherence presents a primary schizophrenia biomarker [46].

In contrast to the current study’s coherence findings implicating reduced connectivity with both posterior temporal regions, the FMAER data demonstrate normal primary receptive auditory processing in the cortex of both STG. This finding stands in contrast with earlier reports, as summarized in Methods, that in some patients with the Landau-Kleffner syndrome [68] and regressive autism [69], the FMAER may be absent and/or distorted. A likely interpretation of the current results is that, in CHR, there is normal initial/early cortical processing of the auditory inputs within the STG but that the STG regions manifest impaired connectivity with other cortical areas that are broadly important in higher level language processing. Putative auditory processing dysfunction in CHR may depend more upon altered access of other regions to the posterior temporal regions and less on language processing within the STG itself – where, in contrast, the defects appear to exist in Landau-Kleffner syndrome and regressive autism.

Our findings of widespread alteration of cortical connectivity in schizophrenia prodrome augment recent findings in animal models of schizophrenia that emphasize abnormalities of functional interactions among various thalamic, frontal, and temporal regions [123–125]. Although progress has been made in avoidance of eye and muscle contamination within quantitative EEG studies, there remain fundamental issues with cleanly accessing and quantifying ‘true’ brain generated delta and gamma band signals that appear most relevant in animal studies which record directly from brain. The process of scalp EEG artifact avoidance no doubt diminishes or degrades true brain delta and gamma signals. Accordingly, current findings cannot exclude potentially relevant delta and gamma coherence differences in CHR. However, current data provide robust indications of a statistically significant CHR cortical difference in CHR at frequencies outside the delta and gamma bands. More work needs to be performed to obviate continuing technical hurdles in processing scalp-recorded EEG.

It must also be emphasized that EEG-based evidence of altered cortical connectivity in CHR should not be considered causal per se. The origins of CHR are likely to involve environmental, developmental, anatomical, genetic, and/or neurochemical factors yet to be conclusively elucidated. Such factors may bring about the behavioral syndrome known as CHR and its associated, potentially identifying, EEG coherence changes.

## Conclusion

This study constitutes a demonstration that, following careful artifact management and objective variable number reduction while maximizing information retention, waking EEG significantly separates patients with CHR (prodromal) schizophrenia from neurotypical controls. The importance of these results rests partly on the relative simplicity and low cost nature of ambient awake EEG data recordings in comparison to the higher cost and complexity of MRI and fMRI data collection processes.

It is also to be noted that EEG data, including passive, specialized auditory stimulation (FMAER), provide additional information – when processed as show above – that augment fMRI-based findings. Current data reinforce prior fMRI findings of temporal lobe dysfunction in schizophrenia. Current EEG data further suggest that auditory hallucinations and auditory processing abnormalities may indeed reflect abnormal connectivity with the posterior temporal lobes but not with associated auditory processing dysfunction within the bilateral superior temporal gyri themselves.

In addition to successfully classifying individual subjects, the canonical discriminant function might also serve as a quantitative biomarker. It is speculated that potentially useful therapeutic interventions might be identified by their effectiveness in moving individual subjects from their initial position along the canonical (biomarker) axis (Fig. 4) as determined by a pre-treatment study, towards the neurotypical end of the axis on the basis of a second post-treatment study following therapeutic intervention. Furthermore, it is speculated that the initial position along the biomarker axis might serve to identify those subjects with prodrome syndrome who are destined to convert to full psychosis/schizophrenia.

However, further studies on larger populations will be required to confirm the demonstrated classification success as well as to establish these hypothetical possibilities regarding use of the discriminant function as a scaled biomarker.

## Abbreviations

BCH: Children’s Hospital Boston
CHR: Schizophrenia prodrome syndrome (clinical high risk) group
CON: Neurotypical control subject group
DFA: Discriminant function analysis
EEG: Electroencephalogram
Fac: Factor, from PCA
FFT: Fast Fourier transform, used for spectral analysis
FMAER: Frequency modulated auditory evoked response
fMRI: functional MRI
iGBR: induced gamma-band EEG response
IRB: Institutional review board
MRI: Magnetic resonance imaging
n/s: not statistically significant
NAPLS-2: Boston site for the North American Prodromal Longitudinal Study
PCA: Principal components analysis
SIPS: Structured Interview for Prodromal Syndromes
SOPS: Scale of Prodromal Symptoms
STG: Superior temporal gyrus/gyri
